# Long-term outcome of uniport vs. multiport video-assisted thoracoscopic lobectomy for lung cancer

**DOI:** 10.1038/s41598-024-55737-8

**Published:** 2024-03-04

**Authors:** Yingding Ruan, Wenjun Cao, Hongsheng Xue, Maoduan You, Zhilong Zhao

**Affiliations:** 1 Department of Thoracic Surgery, The First People’s Hospital of Jiande, JianDe, 311699 China; 2 Department of Thoracic Surgery, Affiliated Zhongshan Hospital of Dalian University, 6 Jiefang Street, Zhongshan District, Dalian, 116044 China

**Keywords:** Uniportal, Multiportal, Video-assisted thoracoscopic surgery (VATS), Lung cancer, Survival time, Respiratory tract diseases, Oncology

## Abstract

This study aims to compare the perioperative outcomes and long-term survival of U-VATS lobectomy for NSCLC with multiportal VATS (M-VATS, involving two ports or more) lobectomy. A total of 339 patients who underwent intentional VATS lobectomy for lung cancer between 2012 and 2017 were included in the analysis. Perioperative outcomes and long-term survival were evaluated. Propensity score matching was utilized to minimize baseline characteristic differences between the two groups. Out of the total cases, 17 (5.01%) were converted to open thoracotomy. The conversion rates were 4.96% (7/141) in the U-VATS group and 5.05% (10/198) in the M-VATS group. A total of 322 consecutive patients underwent VATS lobectomy and mediastinal lymphadenectomy. After propensity matching, 106 pairs were obtained, consisting of 83 males and 129 females. Intraoperative bleeding volume, number of retrieved lymph nodes, explored nodal stations, drainage time and volume, and postoperative hospital stay were similar between the two groups. Both groups exhibited comparable morbidity and mortality rates. From the multivariable analysis, there was no significant difference observed in terms of overall survival (OS) and disease-free survival (DFS) between the two patient cohorts. U-VATS demonstrated comparable perioperative outcomes and long-term efficacy to M-VATS. However, further confirmation of these findings is required.

## Introduction

Worldwide, lung cancer remains the leading cause of cancer incidence and mortality, with an estimated 2.1 million new cases and 1.8 million deaths predicted in 2018^1,2^. Non-small cell lung cancer (NSCLC) accounts for 80–85% of all lung cancers. Anatomic pulmonary resection offers a potential cure for the majority of patients with early-stage NSCLC.

Minimally invasive surgery techniques, such as video-assisted thoracoscopic surgery (VATS) anatomical resectionhave been shown to be a feasible and effective way for the treatment of lung cancer^3,4^.VATS is strongly recommended by the National Comprehensive Cancer Network (NCCN) for NSCLC patients with no surgical contraindications, as long as there is no compromise of oncologic resection principles of thoracic surgery^5^.

After the first case of uniportal VATS (U-VATS) lobectomy was successfully performed in 2011 by Gonzalez et al. U-VATS has emerged as a practicable approach to the treatment of lung cancer^6–8^. However, few studies have reported the long-term results of U-VATS lobectomy for NSCLC until now. This propensity-matched study aimed to compare the long-time survival and perioperative outcomes of U-VATS lobectomy and M-VATS lobectomy for NSCLC.

## Materials and methods

The study was approved by the Ethics Committee of Zhongshan Hospital of Dalian University, and patient consent was obtained to allow collection and analysis of any relevant data. Due to the retrospective data used in this study erasing the patient's name, there was no issue of infringing on patient privacy, and therefore it was exempted by the Ethics Committee.

Data from primary lung cancer patients undergoing intentional VATS lobectomy from January 2012 to December 2017 in the Department of Thoracic Surgery, Zhongshan Hospital of Dalian University, were collected. Preoperative examinations including laboratory tests, electrocardiography, radioisotope bone scanning, pulmonary function tests, chest and abdomen computed tomography, and brain magnetic resonance imaging or computed tomography (CT) were performed for curative surgery. Some patients underwent positron emission tomography-CT (PET-CT) and bronchoscopy.

The inclusion criteria for VATS lobectomy were as follows:

(i) Diagnosis of primary lung cancer.
(ii) No previous history of chest surgery.

The exclusion criteria for VATS lobectomy were as follows:

(i) Transfer to thoracotomy during surgery.

### Surgical technique

All VATS procedures were performed with double-lumen endotracheal intubation, and the patients were positioned in the lateral decubitus position.

For U-VATS or two-port VATS, the surgeon and the second assistant stood on the abdominal side of the patient, while the first assistant holding the camera stood on the other side. A 3 ~ 4 cm incision was made at the 4th or 5th intercostal space on the anterior axillary line. A soft plastic wound protector was applied to the incision without the use of a rib retractor. Another 1–2 cm observation-port was made at the 6th or 7th intercostal space on the mid-axillary line to introduce a 10-mm trocar for two-port VATS.

For three-port VATS, the surgeon and the first assistant usually stood on the abdominal side of the patient, while another assistant stood on theotheropposite side. A 1 cm observation-port was made at the 6th or 7th intercostal space on the mid-axillary line to introduce the 10-mm trocar. Another 3 ~ 4 cm incision at the 4th or 5th intercostal space on the anterior axillary line was made for manipulation. A third incision of 1 cm was made at the 6th or 7th intercostal space on the posterior axillary line as the accessory-port.

The actual key dissection maneuvers are performed similarly using the uniportal approach compared to the M-VATS. Our main procedural sequence involves the dissection and division of veins, arteries, and bronchi. However, the vein was not always divided first as there is limited evidence suggesting that dividing the vein first leads to better prognosis. The choice of surgical methods depended on various factors including the development of pulmonary fissures, obesity, emphysema, and lymph node status. In cases with poor development of pulmonary fissures, single-direction thoracoscopic lobectomy was predominantly performed.

Mediastinal lymphadenectomy was performed after lobectomy. Postoperatively, the chest tube was removed when no air leakage was observed and the volume of drainage was less than 200 ml per day.

All patients were re-staged according to the eighth edition of the tumor, node, and metastasis (TNM) classification of lung cancer^9^ by the International Association for the Study of Lung Cancer (IASLC).

### Data collection

Demographics and clinicopathologic features of the patients, including gender, age, smoking history, resection sites, number of lymph nodes retrieved and nodal stations explored, severe pleural symphysis, operating time, intraoperative bleeding volume, drainage time and volume, postoperative hospital stay, postoperative complications, pathological types and stages, overall survival (OS) and disease-free survival (DFS) were retrospectively collected.

Postoperative complications were defined as deviation from the normal postoperative course within 30 days after surgery, such as pneumonia, atelectasis, delayed wound healing, chylothorax, and prolonged air leak (8 days or longer) in this study. Postoperative 30-day mortality was recorded.

OS was defined as the time from the date of diagnosis until death from any cause, or March 2023. DFS was defined as the time from the date of diagnosis to first progression (locoregional or distant) or death from any cause, or March 2023.

### Statistical analysis and propensity score matching

In order to enhance the comparability and reduce the bias between the two groups, a propensity score analysis with one-to-one matching was performed. The propensity scores were estimated using a logistic model including the following variables: sex, age, smoking, pathological types, and stages.

Measurement data were expressed as mean and standard deviation. Comparisons of categorical data between the two groups were made by using the χ^2^ or Fisher exact test. Continuous data were compared using the Student’s T-test. Statistical analysis was considered to be significant when the probability value was < 0.05.

Univariable analysis of OS and DFS was conducted by a Kaplan–Meier plot, and survival comparisons between the two categories were conducted using log-rank tests. Variables that were significant in univariable analysis (p < 0.05) were included in multivariable analysis to identify independent prognostic factors. Cox proportional hazards regression was used to identify independent prognostic factors in a multivariate analysis.

All of the data analysis was performed using the GraphPad Prism 8 and the Statistical Package for the Social Science software (version 24.0; SPSS Inc, Chicago, IL).

### Ethical approval

The Ethics Committee of Zhongshan Hospital Affiliated with Dalian University and The First People’s Hospital of Jiande approved this study, and the human data was in accordance with the Declaration of Helsinki in the manuscript. Informed consent of patients was waived by The Ethics Committee of Zhongshan Hospital Affiliated to Dalian University.

## Results

### Patient characteristics and perioperative results

**Figure 1:**
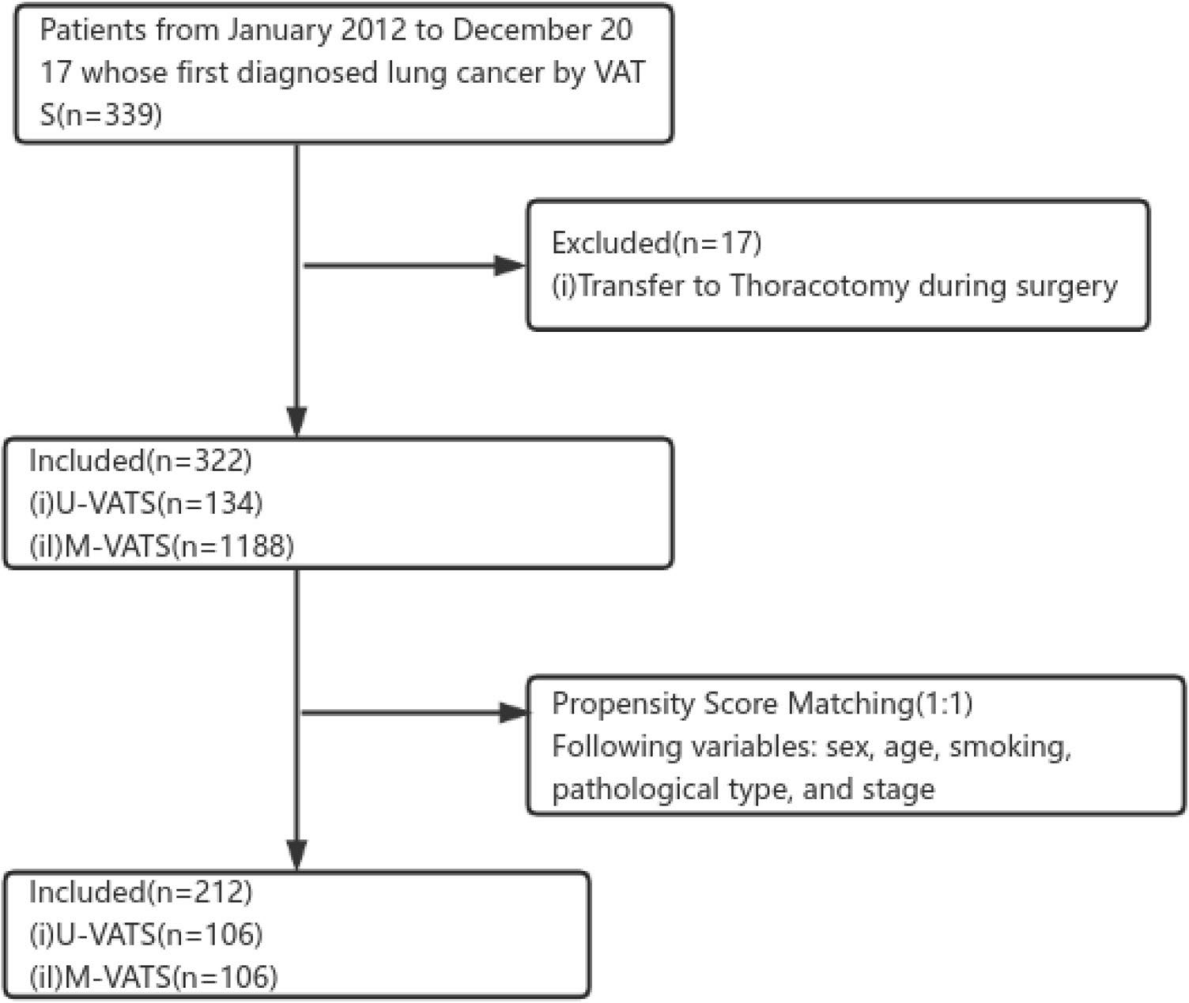
Flow diagram showing schema of study selection of patients with lung cancer (*U-VATS* Uniportal Video-assisted Thoracoscopic Surgery; *M-VATS* Multiportal Video-assisted Thoracoscopic Surgery).

From January 2012 to December 2017, 339 patients underwent intentional VATS lobectomy. Seventeen VATS procedures were converted to open thoracotomy (Fig. 1). The rates of conversion were 4.96% (7/141) in U-VATS and 5.05% (10/198) in M-VATS. Reasons for conversion included severe pleural symphysis in 13 (76.5%) patients and intraoperative bleeding in 4 (23.5%, 1 in U-VATS, and 3 in M-VATS) patients.

Three hundred and twenty-two consecutive patients underwent VATS lobectomy and mediastinal lymphadenectomy. There were 134 patients in the U-VATS group and 188 patients in the M-VATS group. In total, 44 patients had severe pleural adhesions. Before propensity matching, there were 19 patients with severe pleural adhesions in the U-VATS group and 25 patients in the M-VATS group. After matching, there were 12 patients with severe pleural adhesions in each group. All the unmatched information was summarized in Tables 1 and 2.

**Table 1:** Categorical data analysis of the two groups before propensity matching.

Categorical data	U-VATS (n = 134)	M-VATS (n = 188)	*P*-value
Sex (n, %)			0.093
Male	46 (34.3)	82 (43.6)	
Female	88 (65.7)	106 (56.4)	
Smoking (n, %)			0.588
Yes	30 (22.4)	47 (25.0)	
No	104 (77.6)	141 (75.0)	
Postoperative complications (n, %)			0.751
Yes	9 (6.7)	11 (5.9)	
No	125 (93.3)	177 (94.1)	
Pathological Stage* (n, %)			< 0.001
Stage 0	24 (17.9)	25 (13.3)	
Stage I	98 (73.1)	107 (56.9)	
Stage II	5 (3.7%)	21 (11.2)	
Stage III	6 (4.5)	30 (16.0)	
Stage IV	1 (0.7)	5 (2.7)	
Pathological types (n, %)			0.471
Adenocarcinoma	127 (94.8)	170 (90.4)	
Squamous cell carcinoma	5 (3.7)	14 (7.4)	
Adenosquamous	0 (0)	1 (0.5)	
Carcinoid tumors	0 (0)	1 (0.5)	
Small cell carcinoma	2 (1.5)	2 (1.1)	
Status (n, %)			0.01
Death	3 (2.2)	17 (9.0)	
Living	131 (97.8)	171 (91.2)	
Lobectomy (n, %)			0.418
Right upper	59 (44.0)	65 (34.6)	
Right middle	7 (5.2)	10 (5.3)	
Right lower	21 (15.7)	35 (18.6)	
Left upper	34 (25.4)	50 (26.6)	
Left lower	13 (9.7)	28 (14.9)	

*SD* standard deviation.

*8th edition TNM stage grouping.

**Table 2:** Continuous data analysis of the two groups before propensity matching.

Continuous data (Mean ± SD)	U-VATS (n = 134)	M-VATS (n = 188)	*P*-value
Age (year)	59.88 ± 8.74	62.1 ± 10.28	0.022
BMI(kg/m^2^)	24.78 ± 3.30	24.19 ± 3.27	0.115
Operative time (min)	160.83 ± 71.62	180.67 ± 87.56	0.05
Intraoperative bleeding volume (mL)	50.37 ± 46.13	85.18 ± 115.97	< 0.001
Drainage time (days)	4.30 ± 2.75	4.23 ± 2.83	0.841
Drainage volume (ml)	899.44 ± 699.87	965.78 ± 699.60	0.122
Postoperative hospital stay (days)	6.35 ± 2.74	6.96 ± 2.63	0.011
Mediastinal lymph node stations explored	3.81 ± 1.83	3.84 ± 1.99	0.651
Number of lymph nodes retrieved	10.79 ± 7.18	10.37 ± 6.78	0.754

*SD* standard deviation.

After matching, 212 patients (106 couples) were suitable for the analysis (Fig. 1). Intraoperative bleeding volume, operative time, number of lymph nodes retrieved and nodal stations explored, drainage time and volume, the length of postoperative hospital stay, and perioperative complications did not differ between the two groups. Perioperative results of the two groups were reported in Tables 3 and 4.

**Table 3:** Categorical data analysis of the two groups after propensity matching.

Categorical data	U-VATS (n = 106)	M-VATS (n = 106)	*P*-value
Sex (n, %)			0.673
Male	40 (37.7)	43 (40.6)	
Female	66 (62.3)	63 (48.8)	
Smoking (n, %)			0.62
Yes	25 (23.6)	22 (20.8)	
No	81 (76.4)	84 (79.2)	
Postoperative complications (n, %)			0.517
Yes	6 (5.7)	4 (3.8)	
No	100 (94.3)	102 (96.2)	
TNM Stage* (n, %)			0.772
Stage 0	14 (13.2)	20 (18.9)	
Stage I	80 (75.5)	73 (68.9)	
Stage II	5 (4.7)	7 (6.6)	
Stage III	6 (5.7)	5 (4.7)	
Stage IV	1 (0.9)	1 (0.9)	
Pathological types (n, %)			0.375
Adenocarcinoma	102 (96.2)	101 (95.2)	
Squamous cell carcinoma	4 (3.8)	5 (4.8)	
Status (n, %)			0.813
Death	8 (7.5)	9 (8.5)	
Living	98 (92.5)	97 (91.5)	
Lobectomy (n, %)			0.85
Right upper	43 (40.6)	41 (38.7)	
Right middle	6 (5.7)	6 (5.7)	
Right lower	15 (14.2)	20 (18.9)	
Left upper	31 (29.2)	26 (24.5)	
Left lower	11 (10.4)	13 (12.3)	

*8th edition TNM stage grouping.

**Table 4:** Continuous data analysis of the two groups after propensity matching.

Continuous data (Mean ± SD)	U-VATS (n = 106)	M-VATS (n = 106)	*P*-value
Age (year)	60.54 ± 8.79	61.48 ± 10.68	0.421
BMI(kg/ m^2^)	24.67 ± 3.26	24.76 ± 3.75	0.947
Operative time (min)	168.51 ± 76.97	158.09 ± 74.24	0.343
Intraoperative bleeding volume (mL)	53.77 ± 50.45	55.22 ± 54.29	0.633
Drainage time (days)	4.39 ± 2.55	4.07 ± 2.31	0.209
Drainage volume (ml)	952.97 ± 716.73	904.50 ± 591.88	0.876
Postoperative hospital stay(days)	6.56 ± 2.63	6.24 ± 2.39	0.233
Mediastinal lymph node stations explored	3.81 ± 1.62	3.86 ± 2.04	0.466
Number of lymph nodes retrieved	10.99 ± 7.06	11.09 ± 7.51	0.744

*SD* standard deviation.

The main complications were air leakage and pulmonary infection. There were 10 patients with prolonged air leakage, 6 patients recovered with no further procedure, 2 healed after negative pressure suction, and 2 healed after 50% glucose injection in the pleural cavity. In both groups, the 30-day mortality rate was 0%; no patient suffered from arrhythmia for more than 1 day, as well as surgical reoperation for postoperative hemorrhage.

### Survival analysis

**Figure 2:**
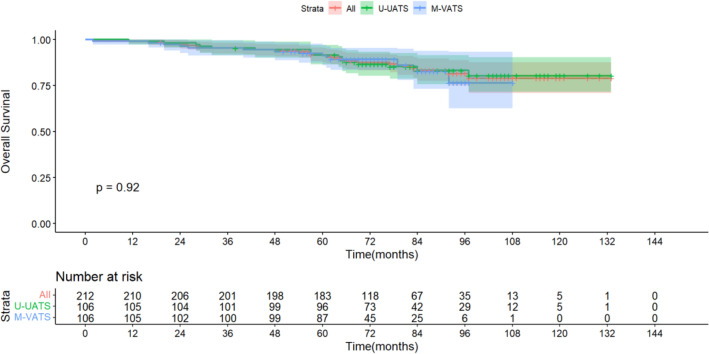
Overall Survival.

**Figure 3:**
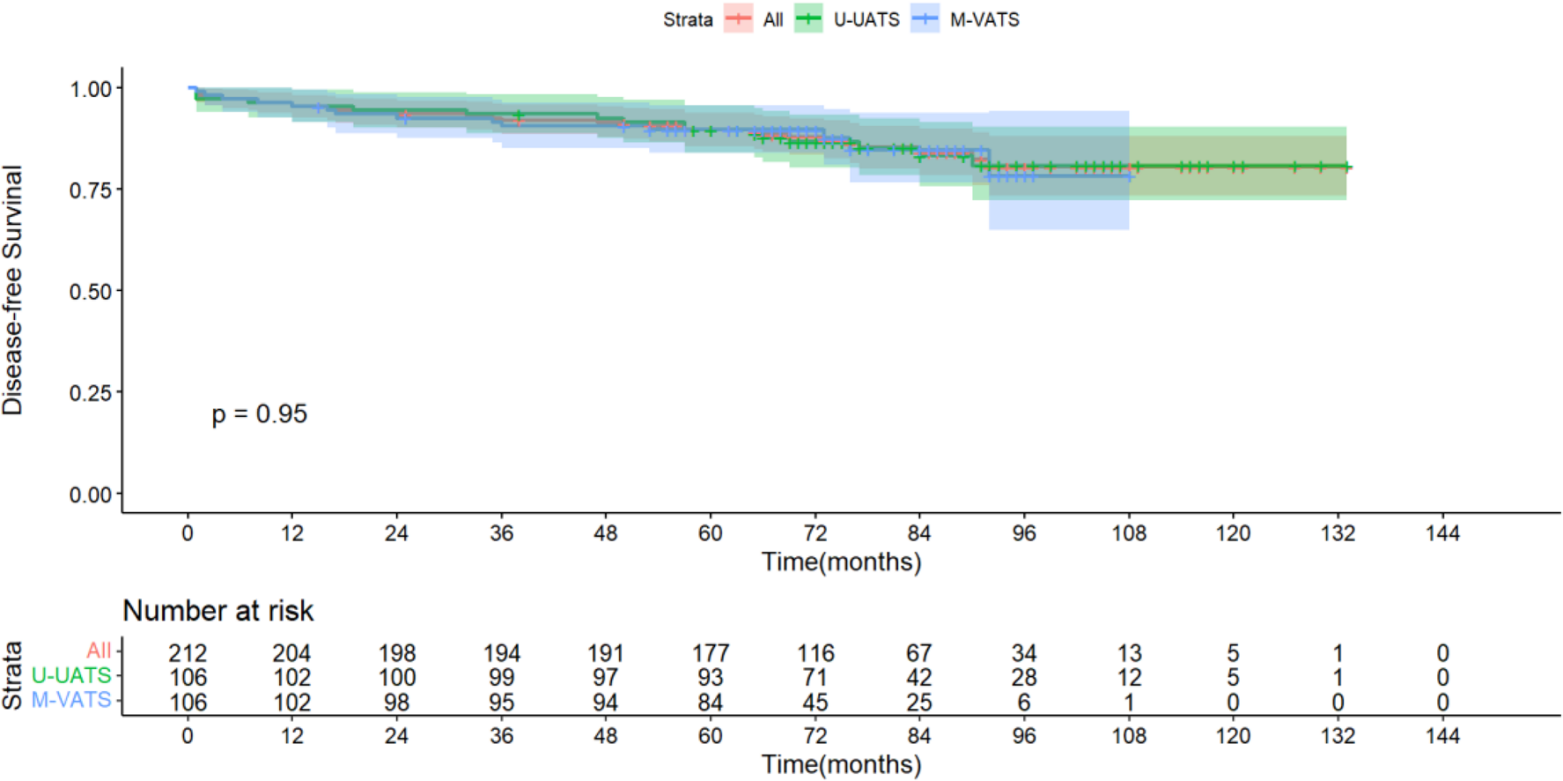
Disease-Free Survival.

After matching, the average DFS of patients undergoing U-VATS and M-VATS was (67.33 ± 2.160) months and (81.61 ± 2.805) months respectively (P = 0.951). The average OS of patients undergoing U-VATS and M-VATS was (69.69 ± 1.780) months and (81.97 ± 2.183) months respectively (P = 0.917) (Figs. 2 and 3).

Both U-VATS and M-VATS presented similar outcomes on long-term survival. In fact, no significant difference was reported between the two groups regarding the OS and DFS at 5 years (89.2% vs. 86.5%) and (89.5% vs. 89.6%).

Using univariable analysis, we found the following factors to be associated with the OS and DFS: Age, Sex, TNM stage, Drainage time, Drainage volume, Postoperative hospital stay, and Number of lymph nodes retrieved, which was summarized in Table 5. In the multivariable analysis, only the TNM stage was an independently associated factor to OS (Hazard ratio (HR): 2.793, 95% confidence interval (CI) 1.848–4.219) and DFS (Hazard ratio (HR): 2.972, 95% confidence interval (CI) 1.934–4.564), which was summarized in Table 6.

**Table 5:** Univariate analysis affecting OS and DFS of lung cancer patients.

Variables	OS				DFS			
	*P*-value	Hazard ratio (HR)	95% Exp(B) CI		*P*-value	Hazard ratio (HR)	95% Exp(B) CI	
			Down	Up			Down	Up
Surgical approach	0.922	1.037	0.505	2.129	0.951	0.978	0.479	1.997
Age	0.046	1.04	1.001	1.081	0.051	1.039	1	1.079
Sex	0.017	0.415	0.201	0.855	0.016	0.441	0.199	0.846
Pathological types	0.69	1.34	0.319	5.628	0.696	1.331	0.317	5.593
TNM Stage	< 0.01	2.513	1.739	3.361	< 0.01	2.563	1.779	3.692
Lobectomy	0.292	1.135	0.897	1.437	0.266	1.143	0.903	1.446
Smoking	0.096	0.532	0.253	1.118	0.098	0.536	0.255	1.122
Postoperative complications	0.174	0.438	0.133	1.442	0.162	0.428	0.13	1.408
BMI	0.827	0.988	0.886	1.102	0.826	0.988	0.885	1.102
Operative time	0.599	1.001	0.997	1.006	0.584	1.001	0.997	1.006
Intraoperative bleeding volume	0.528	0.997	0.987	1.007	0.524	0.997	0.987	1.007
Drainage time	0.018	1.113	1.019	1.216	0.015	1.117	1.022	1.222
Drainage volume	0.001	1.001	1	1.001	< 0.01	1.001	1	1.001
Mediastinal lymph node stations explored	0.379	1.093	0.896	1.334	0.339	1.102	0.903	1.344
Number of lymph nodes retrieved	0.042	1.045	1.001	1.091	0.035	1.047	1.003	1.093
Postoperative hospital stay	0.043	1.109	1.003	1.226	0.043	1.111	1.003	1.231

**Table 6:** Multivariable analysis affecting OS and DFS of lung cancer patients.

Variables	OS				DFS			
	*P*-value	Hazard ratio (HR)	95% Exp(B) CI		*P*-value	Hazard ratio (HR)	95% Exp(B) CI	
			Down	Up			Down	Up
Age	0.099	1.037	0.993	1.082	0.095	1.038	0.994	1.083
Sex	0.099	0.523	0.242	1.13	0.092	0.516	0.24	1.113
TNM stage	< 0.01	2.793	1.848	4.219	< 0.01	2.972	1.935	4.564
Drainage time	0.401	0.876	0.643	1.193	0.313	0.851	0.621	1.165
Drainage volume	0.086	1.001	1	1.001	0.062	1.001	1	1.001
Number of lymph nodes retrieved	0.857	1.004	0.962	1.048	0.811	1.005	0.962	1.051
Postoperative hospital stay	0.351	1.132	0.873	1.467	0.298	1.147	0.886	1.486

Surgical approach*: U-VATS vs M-VATS.

## Discussion

Since 1992, VATS has been increasingly utilized for treatment of the NSCLC. Because of the development of novel surgical techniques and instruments, VATS lobectomy for early-stage NSCLC patients has become more and more common. Within the European Society of Thoracic Surgeons (ESTS) database of approximately 40,000 lobectomies, the percentage of VATS lobectomy was 31.9 between 2013 and 2017; this percentage was only 5.3 between 2007 and 2012^10^.

With increased VATS lobectomy cases and gained experience, more and more surgeons consider U-VATS as a feasible option^5,6,8^. In 2013, the first U-VATS lobectomy was completed in our institution. The proportion of U-VATS lobectomy gradually increased to 29.4% (20 / 68) by 2016 and 82.9% (87/105) by 2017. At present, U-VATS has become the most commonly used method for surgical resection of NSCLC in this hospital. The rates of conversion were 4.96% (7/141) in U-VATS and 5.05% (10/198) in M-VATS.

Compared with M-VATS, the advantages of U-VATS include fewer incisions, less pain, shorter hospital stays, and faster recovery of lung function^11–13^. There are also some disadvantages of U-VATS, such as prolonged time when performing lymph node dissection, especially for subcarinal lymph nodes. Also, because of the small space, the surgical instruments and the thoracoscope will interfere with each other. On the other hand, the assistant holding the camera may be prone to fatigue if the operation time is too long. According to our experience, these limitations are obvious in the early practicing stage but can be well overcome by skilled surgeons.

With the accumulation of experience, many complicated and difficult U-VATS procedures, such as bronchoplasty, tracheal resection, carinal resection, and reconstruction, lobectomies with en bloc chest wall excision, vascular reconstruction, and esophagectomy, have been reported^3,13,14^. However, conventional thoracotomy is still the most widely used approach in complicated cases and extensive resection considering the safety and the principles of an oncologic resection.

One point of concern is the operation time with the U-VATS procedure. A multicenter retrospective cohort study reported that 458 patients (166 patients in the U-VATS group and 292 patients in the M-VATS group) were enrolled. Operation time in the U-VATS group was significantly longer (171.6 ± 36.3 min vs. 162.4 + 51.0 min), but the demographics and clinicopathologic features were not significantly different^15^. However, other studies showed no significant difference in operation time between U-VATS and M-VATS^16–18^.

The operation time of the U-VATS group in this study was significantly shorter (160.83 min) than that of the M-VATS group (180.67 min) before propensity matching. After propensity matching, the operation time of U-VATS group was slightly longer (168.51 min vs. 158.09 min, P = 0.343).

Another concern is the risk of blood loss from U-VATS lobectomy and mediastinal lymphadenectomy. Both before and after propensity matching in this study, intraoperative bleeding was significantly different between the two cohorts. This was confirmed by other studies. Dai et al. reported in a propensity-matched study that 63 patients with lung cancer who underwent U-VATS had less intraoperative bleeding, less pain, and higher satisfaction scores than the patients undergoing two-port VATS^17^. A meta-analysis of 11 studies showed that patients in the uniportal group had a significant reduction in the duration of postoperative drainage (uniportal: 4.39 days vs. multiportal: 4.99 days; P = 0.003), bleeding volume (97.7 ml vs. 116.7 mL; P = 0.006), length of hospital stay (6.3 days vs. 7.0 days; P < 0.001), postoperative pain (2.53 vs. 4.22, P = 0.02), and complication rate (14.5% vs. 17.5%; P = 0.008). There were no significant differences between the two groups with regards to mortality, operative time, number of dissected lymph nodes, and conversion rate^18^.

Wang et al. reported that in their experience with 257 patients undergoing VATS lobectomy including 73 patients in the uniportal group, 86 in the two-port group, and 98 in the three-port group, there were no significant differences in intraoperative bleeding, operation time, number of lymph nodes retrieved and nodal stations explored, drainage times and volume, and postoperative hospital stay among the three groups. The study concluded that the pain score in the U-VATS group was significantly reduced^19^.

Chang et al. reported that postoperative hospital stay after uniportal and two-port VATS were 5 days and 6 days respectively^17^. Similarly, a propensity-matched study showed the length of postoperative hospital stay was 4.7 days in U-VATS and 5.3 days in M-VATS^20^.

In this study, postoperative hospital stay after uniportal and multiport VATS were respectively 6.35 days and 6.96 days (p = 0.011) before matching, 6.56 days and 6.24 days (p = 0.233) after matching. U-VATS lung cancer resection does not prolong postoperative hospital stay compared with M-VATS.

Complications have always been a safety concern, especially for catastrophic events. In this study, there was no significant difference in the incidence of postoperative complications between the two cohorts before and after matching. Similar findings were reported recently^6,17,19,20^.

Rates of conversion from VATS to open thoracotomy ranging from 0 to 23% have been reported, which might be caused by bleeding and severe pleural symphysis^19,21,22^. Gonzalez-Rivas et al.^23^ reported that pleural symphysis was a predictor of complications. Patients with pleural symphysis had a higher postoperative complications rate (31.5% vs. 14.5%, P < 0.001). At the same time, pleural adhesions can affect postoperative recovery. In our series, in patients who presented with pleural adhesions difficult to negotiate, the VATS procedure was straightly converted to thoracotomy due to safety and efficacy concerns. In all of the cases, there was no sleeve lobectomy.

As the study shows, there are no significant differences between the two cohorts with regard to a number of lymph nodes retrieved, nodal stations explored, drainage time and volume, and postoperative complications. U-VATS is comparable to M-VATS in terms of safety and effectiveness, which is the same as previous reports^8,24,25^.

Our survival analysis showed that OS and DFS of the two groups were comparable, and surgical methods (U-VATS and M-VATS) were not the absolute risk factors for DFS (p > 0.05) or OS (p > 0.05) in NSCLC patients. This result is similar to that of previous related studies^26,27^. Both results show that the number of incisions does not affect the long-term survival of patients, because it is unlikely to be related to TNM staging or pathological progress of lung cancer. The incision usually affects postoperative recovery. Therefore, whether the survival rate of U-VATS is better than that of M-VATS needs further and larger randomized controlled trials.

Accurate TNM staging is an important prerequisite for selecting treatment methods and evaluating prognosis. At present, a consensus has been reached on the surgical treatment including VATS for stage I and stage II of NSCLC, but the choice of treatment methods for stage IIIA and above NSCLC is still controversial^28^. According to some studies, for patients with stage IIIA or above, especially for patients with oligometastatic, VATS lobectomy can still be performed, and patients are more likely to have a better prognosis^2,3^. In this study, there were 36 patients with stage III and 6 patients with oligometastasis of stage VI. After matching, only 13 patients were in the two groups (11 patients in stage III and 2 patients in stage VI). The 5-year overall survival rate of stage III patients is as high as 80–90%, and that of stage VI patients is 30–40%. Cox multivariate analysis showed that the TNM stage was an independent prognostic factor of NSCLC.

The varied learning curves associated with U-VATS and M-VATS, along with differences in surgical experience, could potentially impact patient outcomes. The authors, with more than ten years of thoracotomy experience, have been performing M-VATS lobectomy since 2001. After 3 years of performing two-ports VATS lobectomy, we began U-VATS lobectomy in 2013. Based on our experience, it is recommended to have 30–50 cases of M-VATS lobectomy before undergoing U-VATS lobectomy for NSCLC.

The operative technique is well defined for the different lobectomies and for the mediastinal lymphadenectomy. The parallel instrumentation achieved during the single port approach mimics the inside maneuvers performed during open surgery, together with the direct view facilitates the dissection and division of the hilar structures and the fissure. This makes possible the direct transition from open surgery to uniportal VATS. Uniportal VATS is feasible and reproducible. This is why its use is spreading in many centers in Spain, Europe and Asia, with good results^26,27,29^.

There are several limitations in this study. First, the number of cases of U-VATS for NSCLC has gradually increased, and it has become the most common procedure since 2016. Most M-VATS cases in this cohort were completed early. Second, acute or chronic pain analysis was not performed. Thirdly, this study is a retrospective study with inevitable selection bias even with PSM conducted. Finally, this study is a single-center study lacking diversity. Therefore, more exploration through multicenter prospective studies is needed.

## Conclusion

Despite the intrinsic limitations of this study, it can be concluded that U-VATS may be feasible and safe for anatomical resection of NSCLC compared with M-VATS. U-VATS is not inferior to M-VATS in surgical outcomes or long-term prognosis in patients with lobectomy of NSCLC. Further studies based on larger populations with long-term follow-up are required to determine its further benefits to patients.

## Data Availability

You can contact the first author to obtain the original data: Yingding Ruan. Email: ruanyingding@sina.com.

## Abbreviations

U-VATS: Uniportal video-assisted thoracoscopic surgery
M-VATS: Multiportal video-assisted thoracoscopic surgery
NSCLC: Non-small cell lung cancer
PET: Positron emission tomography
CT: Computed tomography
DFS: Disease- free survival
OS: Overall survival
